# Inequalities in adolescent HPV, Td/IPV and MenACWY vaccination coverage by socio-economic status: an ecological study, England, 2017 to 2024

**DOI:** 10.2807/1560-7917.ES.2026.31.13.2500586

**Published:** 2026-04-02

**Authors:** Muhammad Ibaad Alvi, Laura Viviani, Susan Hopkins, Kate Soldan, Sharif Ismail

**Affiliations:** 1 United Kingdom Health Security Agency, London, United Kingdom; * These authors contributed equally to this work and share last authorship.

**Keywords:** vaccination programme, MenACWY, HPV, TdIPV, adolescent, school-based programme, cervical, oropharyngeal

## Abstract

**BACKGROUND:**

Many countries use school-based vaccination for adolescent vaccination; it has been shown to reduce inequities in uptake compared with other delivery routes. In England, coverage for human papillomavirus (HPV), meningococcal groups A, C, W and Y (MenACWY) and tetanus, diphtheria and inactivated polio virus (Td/IPV) vaccine programmes exceeded 80% before the COVID-19 pandemic. However, recent data show declining uptake.

**AIM:**

This study examined the relationship between socio-economic deprivation and coverage over time.

**METHODS:**

We conducted an ecological analysis of first-dose coverage for HPV (females and males), MenACWY and TdIPV in adolescents across 150 local authorities in England from 2017 to 2024. Coverage data were linked to 2025 Index of Multiple Deprivation (IMD) scores. Associations between IMD quintile, academic year and vaccination coverage were estimated using beta regression models.

**RESULTS:**

Between 2020 and 2024, lower coverage was consistently associated with higher deprivation, and differences in coverage between the most and least deprived IMD quintiles more than doubled. In 2024 these differences were 17.7%, 18.2%, 16∙8% and 16.9% for HPV (females), HPV (males), MenACWY and Td/IPV, respectively. The consistency of these findings suggests the effect of deprivation on coverage is not vaccine-specific.

**CONCLUSION:**

We demonstrated a consistent and strengthening association between coverage and deprivation across multiple adolescent school-based vaccination programmes in England over time. Contributory factors may be numerous, and further research is needed to understand which factors are driving trends for different populations. Addressing these inequalities will require sustained targeted interventions to improve awareness of and access to vaccination.

**Key public health message**

**What did you want to address in this study and why?**

Vaccines given in schools help protect children and teenagers from a variety of infections and some cancers. Before COVID-19, vaccination coverage was high across England, with similar rates in more and less deprived areas but has decreased since. We sought to understand if differences between more and less deprived communities have emerged in three vaccines delivered to adolescents: HPV (males and females), MenACWY, and TdIPV.

**What have we learnt from this study?**

Since 2020, coverage has decreased across all three programmes, and a clear gap has emerged where teenagers living in more deprived areas are now less likely to be vaccinated than those is less deprived areas. These gaps have more than doubled over time and are seen across all three vaccines, which suggests that there is a wider problem with the programme and its delivery rather than an issue with a single vaccine.

**What are the implications of your findings for public health?**

Lower rates of vaccination in more deprived areas increase the risk of inequalities in the diseases these vaccines prevent. This risks a worsening of differences in overall health between more and less deprived areas. Understanding what is driving the differences in rates of vaccination will be important when designing interventions that address such inequalities.

## Introduction

Adolescent immunisation programmes in England aim to protect young people from vaccine-preventable diseases through school-based delivery of three vaccinations. These are: human papillomavirus (HPV) vaccination which protects against infections with HPV types that can cause cervical cancer, oropharyngeal cancers and anogenital warts, the meningococcal ACWY (MenACWY) vaccination, which protects against invasive meningococcal disease, and a combined vaccination which protects against tetanus, diphtheria and polio (Td/IPV; inactivated polio vaccine) [1]. Of these three, the HPV and MenACWY vaccines are first-dose vaccinations, whereas Td/IPV is a booster.

Before the COVID-19 pandemic, coverage for all three vaccines in England exceeded 80% of all adolescents identified as eligible – exceeding coverage rates observed for adolescent vaccination in many high-income comparator countries, and across the World Health Organization European Region [2-5]. However, there were significant disruptions to delivery during the pandemic associated with a substantial reduction in overall coverage, as for routine programmes in many countries worldwide. While catch-up activities appear to have contributed to a stabilisation in uptake trends since that time, national coverage in 2025 remained well below that observed before the pandemic. There is also evidence of variation in uptake rates for HPV vaccination by sex (since the addition of boys to create a universal HPV programme in 2019) and by geography [3,6,7].

School-based vaccination is now used as a key delivery mechanism in many countries worldwide, and in some European countries [8]. There is good evidence internationally to suggest that school-based delivery reduces inequalities in uptake that are often observed when using other mechanisms [9-11]. In England, the adolescent vaccine programme is delivered in secondary schools. Public health surveillance from the early years of the programme in England, when HPV vaccination was delivered via a mixture of school- and primary care-based routes, showed substantially lower uptake in those areas offering vaccination via the latter [12].

There is increasing international evidence to suggest that HPV vaccination uptake is lower among socio-economically deprived groups – as is observed for many routine vaccination programmes – although this pattern is not universally seen. There is some evidence to suggest, for example, that a more complex picture applies in countries where multi-dose schedules are used [13,14]. In England, a study of uptake data for the period 2008 to 2011 found no significant association between routine HPV vaccination coverage and the index of multiple deprivation (IMD) at the local authority level [15]. However, lower coverage was associated with lower IMD among the older adolescents in the initial catch-up programme that was delivered in education and other settings. Overall, coverage in this catch-up programme was lower than in the routine vaccination programme. Associations between socio-economic status and MenACWY and Td/IPV uptake have not previously been explored in depth, although there is some evidence to suggest that school type and local area deprivation are important factors in MenACWY uptake as they are for HPV [16].

Evidence on uptake of routine childhood immunisations suggests that effects of deprivation on vaccination uptake have intensified in England after the COVID-19 pandemic [17], overlaying known inequalities related to various population factors that pre-dated COVID-19 [18]. However, this had not been explored for adolescent vaccination in England. In addition, important changes in school attendance patterns have been noted since the pandemic, including an increase in pupil absences overall by comparison with the pre-pandemic period, and evidence that these absences are concentrated in areas of greater socio-economic deprivation where timely access to health services is recognised to be more challenging [19].

In view of the changes outlined above, this study aimed to assess the relationship between deprivation and uptake for these three adolescent programmes, and whether – and how – inequalities in vaccination coverage have emerged since the pandemic. We assessed the relationship between the IMD and coverage for HPV, MenACWY, and Td/IPV vaccination across the 150 English local authorities from 2017 to 2024. In doing so, we aimed to inform activities to reduce inequalities in vaccine-preventable disease incidence and strengthen the resilience of the adolescent vaccination offer in England.

## Methods

### Programme context

A national, adolescent HPV immunisation programme began in England in 2008 to protect against cervical cancer, offering routine vaccination to girls during the school year when they turn 13-years-old. This expanded from 2019 to include same aged boys [20]. A catch-up programme was also offered to girls up to 18-years-old in 2008 and 2009. The initial schedule of three doses was reduced to two doses in 2014 and to one dose in 2023. The motivation for expanding to a universal HPV programme in England was to bolster resilience across the vaccination programme overall, to strengthen population immunity to protect unvaccinated girls, and to provide males (including those who have sex with men) with direct protection against the range of HPV-related diseases [20]. Eligible adolescents who miss the routine offer remain eligible until age 25 years.

The MenACWY programme was introduced in 2015 to respond to a rapid increase in cases of invasive meningococcal group W (MenW) disease [21]. The MenACWY vaccine is given to children as a single-dose injection at around 14 years of age. It provides first-time protection against MenA, W and Y and boosts the MenC vaccine given to infants around the age of 1 year. Teenagers who miss having the MenACWY vaccine at 14 years can still receive it up until the age of 25 years.

Finally, the Td/IPV booster, a longstanding component of the adolescent schedule, is delivered alongside MenACWY to maintain population immunity against tetanus, diphtheria, and polio [22].

### Data sources

First-dose coverage data for HPV and for MenACWY and Td/IPV (ages 14–15 years), disaggregated by English upper tier local authority (UTLA) were obtained from the United Kingdom Health Security Agency (UKHSA) statistical publications spanning the seven academic years 2017–18 to 2023–24. We focused on dose 1 coverage for HPV among males and females in school year 9, and coverage in year 10 for both MenACWY and Td/IPV to capture data pertaining to the initial offer (year 8 for HPV and year 9 for MenACWY and Td/IPV) and one year of catch-up activities. Data disaggregated by sex were available only for HPV, in line with local area level reporting in national surveillance systems.

For our analysis, deprivation was quantified using IMD 2025 scores at UTLA level [23]. The IMD scores are used in England as composite, area-level measures of relative deprivation based on the following seven domains: income, employment, education, skills and training, health and disability, crime, access to housing and service, and the local living environment. Areas are ranked from the most deprived area (lowest score) to the least deprived area (highest score). For our analysis, we grouped the 150 UTLAs into quintiles of deprivation (Q1 to Q5, the most deprived quintile being Q1). Changes in the boundaries of UTLAs over the study period meant that six areas could not be matched to a corresponding IMD 2025 score and were therefore excluded from our analysis. These were City of London (a borough within London), West Northamptonshire, North Northamptonshire, Bournemouth, Poole Local Authorities and Cumbria.

We analysed coverage trends in HPV (females – hereafter denoted HPV-F; and males – denoted HPV-M), MenACWY and Td/IPV in 150 UTLAs, from September 2017 to August 2024 for HPV (2020 to 2024 in males) and MenACWY, and from 2016 to 2024 for Td/IPV. Average coverage across all programmes declined after 2019. To adequately capture this downward trend and better reflect the breaking point represented by pandemic-related disruptions to service delivery, we modelled the decline in vaccination coverage from academic year 2019–20 (first cohort affected by the pandemic) onwards.

### Statistical analysis

We computed vaccination coverage at UTLA level by dividing the number of vaccinated individuals by the number of individuals eligible for vaccination.

We used Beta regression to estimate the effect of IMD quintile and academic year on coverage. Beta regression is useful when modelling continuous dependent variables that are bounded between 0 and 1 (such as proportions). To account for the wide variation in population size (ranging from 6 to > 2,000) across local authorities, we weighted regressions by the number of eligible individuals in each UTLA. Finally, we formally tested whether the effect of IMD on vaccination coverage changed over time by including in the model an interaction term between IMD quintile and academic year. Further details are provided in the Supplement.

### Assessment of a London effect

Uptake across all programmes (adolescent, childhood and other) has been consistently lower for London than for other English regions for some years, for reasons including high socio-economic inequality, high population mobility, and complex service delivery arrangements, among others [17]. To assess the extent to which a ‘London effect’ may be influencing our results, we repeated the analysis excluding all boroughs in London and Greater London.

## Results

**Figure 1:**
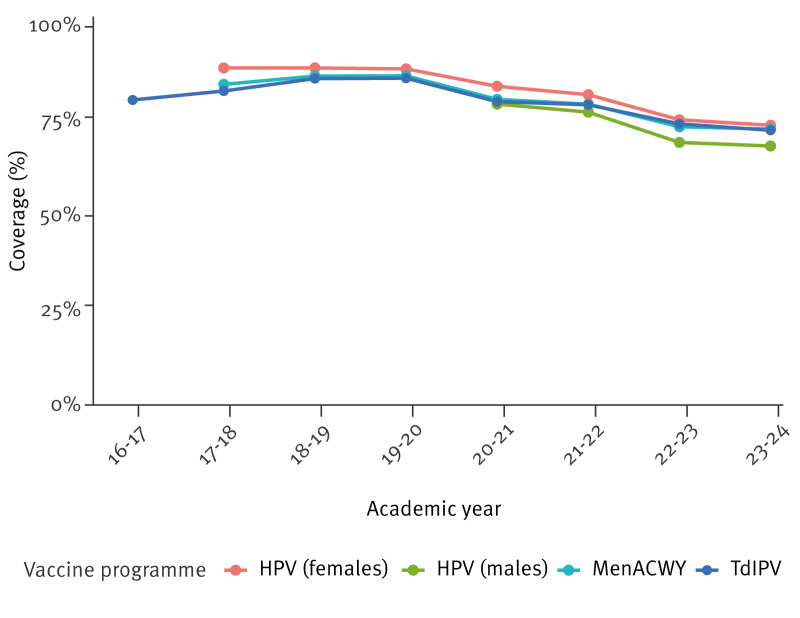
Total vaccine coverage over time, by vaccination programme, England, 2017–2024 (n = 12,823,054)

HPV: human papillomavirus; MenACWY: meningococcal group A, C, W and Y vaccine; TdIPV: tetanus, diphtheria and inactivated polio virus vaccine.

Table 1 shows summary statistics for coverage (national average, and minimum, 25th percentile, 75th percentile and maximum across UTLAs) of the four programmes between 2016 and 2024, and the number of eligible individuals. For each of the four programmes, we observed a decline in coverage over the years. National coverage was highest before the pandemic for all four programmes: for HPV-F at 89.0% in 2017–18, HPV-M at 79.5% in 2020–21, MenACWY at 87.0% in 2019–20 and TdIPV at 86.3% in 2019–20. All programmes then saw a sustained decline in the post-pandemic period. This is demonstrated in Figure 1, where 2023–24 was the overall worst year for coverage: 73.9% for HPV-F, 68.5% for HPV-M, 72.9% for MenACWY and 72.6% TdIPV. The difference between the 75th percentile and 25th percentile, the interquartile range (IQR), is a useful measure of variation of vaccine coverage of the middle 50% of observations. The IQR expanded substantially over the observation period for all four vaccination programmes. The IQR for HPV-F more than doubled from 6.6 percentage points in 2017–18 to 14.7 points in 2023–24; for HPV-M it increased from 12.2 in 2020–21 to 17.1 in 2023–24; for TdIPV it increased from 9.6 in 2016–17 to 16.3 points in 2023–24; for MenACWY it increased from 10.38 points in 2017–18 to 16.0 in 2023–24. The 25th percentile for coverage also fell faster than the 75th percentile for coverage.

**Table 1:** Summary statistics for vaccination coverage across four vaccine programmes, England, 2017–2024 (n = 12,823,054)

Academic year	Number eligible	Coverage (% vaccinated)				
		England overall	Min	25th percentile	75th percentile	Max
HPV-F						
2017–18	291,241	89.04	68.03	86.10	92.67	98.56
2018–19	297,744	89.03	65.70	86.79	92.5	99.20
2019–20	306,913	88.78	64.50	85.70	92.50	98.50
2020–21	303,392	84.20	49.44	80.81	90.39	97.23
2021–22	332,462	81.98	31.70	78.50	87.50	97.30
2022–23	331,791	75.38	38.70	69.50	83.40	93.70
2023–24	338,382	73.93	25.52	68.59	83.25	94.72
**All years**	**2,201,925**	**82.89**	**25.52**	**79.11**	**90.50**	**99.20**
HPV-M						
2020–21	315,259	79.52	42.56	73.83	85.99	100
2021–22	343,867	77.39	34.70	72.50	83.00	99.60
2022–23	343,363	69.40	28.20	60.60	78.90	90.80
2023–24	353,505	68.46	25.81	61.25	78.36	100
**All years**	**1,355,994**	**73.53**	**25.81**	**67.50**	**82.40**	**100**
MenACWY						
2017–18	554,579	84.71	57.52	80.29	90.67	98.43
2018–19	590,570	86.90	60.70	83.00	91.30	97.20
2019–20	606,373	86.95	35.06	84.21	92.58	99.73
2020–21	594,891	80.76	44.60	74.90	86.90	98.70
2021–22	651,686	79.48	48.17	74.34	86.46	100
2022–23	681,147	73.53	29.61	65.69	81.87	96.32
2023–24	687,143	72.90	31.92	65.72	81.72	97.65
**All years**	**4,366,389**	**80.40**	**29.61**	**74.89**	**88.24**	**100**
TdIPV						
2016–17	524,760	80.59	13.57	77.73	87.30	100
2017–18	554,579	82.96	29.62	78.6	90.33	98.15
2018–19	590,570	86.22	63.00	82.20	90.19	97.62
2019–20	606,373	86.33	35.32	83.92	92.09	97.14
2020–21	602,488	80.15	44.96	74.70	86.12	98.03
2021–22	651,686	79.32	48.17	74.29	85.40	99.82
2022–23	681,147	74.28	29.61	66.01	84.48	97.8
2023–24	687,143	72.60	31.89	65.61	81.87	96.81
**All years**	**4,898,746**	**80.03**	**13.57**	**74.86**	**87.77**	**100**

F: females; HPV: human papillomavirus; M: males; MenACWY: meningococcal group A, C, W and Y vaccine; TdIPV: tetanus, diphtheria and inactivated polio virus vaccine.

The results of the beta regression models for each vaccine programme are shown in Table 2. The relationship between coverage and academic year was significant (p < 0.001) across all four programmes. After adjusting for the effect of IMD on vaccine coverage, the effect of academic year was largest for HPV-F (adjusted odds ratio (aOR) of 0.79, indicating a reduction in the odds of coverage by 21.0% for each year), and smallest in TdIPV (aOR of 0.83 indicating a reduction in the odds of coverage by 17% for each year).

**Table 2:** Effect of the index of multiple deprivation and academic year on vaccine coverage, by vaccination programme, England, 2017–2024 (n = 2,788)

Effect	aOR (95% CI)	p value
HPV-F		
IMD quintile (Q2 vs Q1)	1.273 (1.270–1.277)	< 0.001
IMD quintile (Q3 vs Q1)	1.364 (1.361–1.368)	< 0.001
IMD quintile (Q4 vs Q1)	1.561 (1.557–1.565)	< 0.001
IMD quintile (Q5 vs Q1)	1.839 (1.834–1.843)	< 0.001
Academic year	0.786 (0.786–0.786)	< 0.001
HPV-M		
IMD quintile (Q2 vs Q1)	1.266 (1.262–1.269)	< 0.001
IMD quintile (Q3 vs Q1)	1.402 (1.398–1.406)	< 0.001
IMD quintile (Q4 vs Q1)	1.549 (1.545–1.553)	< 0.001
IMD quintile (Q5 vs Q1)	1.905 (1.900–1.910)	< 0.001
Academic year	0.817 (0.817–0.818)	< 0.001
MenACWY		
IMD quintile (Q2 vs Q1)	1.204 (1.202–1.206)	< 0.001
IMD quintile (Q3 vs Q1)	1.340 (1.338–1.343)	< 0.001
IMD quintile (Q4 vs Q1)	1.661 (1.658–1.664)	< 0.001
IMD quintile (Q5 vs Q1)	1.978 (1.974–1.982)	< 0.001
Academic year	0.820 (0.820–0.820)	< 0.001
TdIPV		
IMD quintile (Q2 vs Q1)	1.243 (1.240–1.246)	< 0.001
IMD quintile (Q3 vs Q1)	1.388 (1.386–1.391)	< 0.001
IMD quintile (Q4 vs Q1)	1.739 (1.735–1.742)	< 0.001
IMD quintile (Q5 vs Q1)	2.077 (2.073–2.081)	< 0.001
Academic year	0.826 (0.825–0.826)	< 0.001

CI: confidence interval; F: females; HPV: human papillomavirus; IMD: index of multiple deprivation; M: males; MenACWY: meningococcal group A, C, W and Y vaccine; aOR: adjusted odds ratio; Q1: most deprived; Q5: least deprived; TdIPV: tetanus, diphtheria and inactivated polio virus vaccine.

The aORs are derived from a beta regression model including IMD quintile and academic year. The IMD quintile is modelled as a categorical variable with Q1 (most deprived) as the reference group; aORs represent the relative odds of higher vaccination coverage compared with Q1. Academic year is modelled as a continuous ordered variable (coded 1–5 for 2019–20 to 2023–24); the aOR represents the change in odds of coverage per 1-year increase.

The relationship between coverage and deprivation (IMD quintile) was also significant (p < 0.001) across all four programmes for all years after 2019. The aOR of the effect of IMD quintile increased as IMD quintile increased across each programme, for example for HPV-F, the aORs were 1.27, 1.36, 1.56 and 1.84 for Q2, Q3, Q4 and Q5, respectively (vs Q1). The aOR was largest in TdIPV (OR of 2.08 Q5 vs Q1, indicating 108% higher odds of coverage in the least deprived quintile (Q5) compared with the most deprived), and smallest in HPV-F (aOR of 1.84 indicating 84.0% higher odds of coverage in the least deprived quintile (Q5) compared with the most deprived).

**Figure 2:**
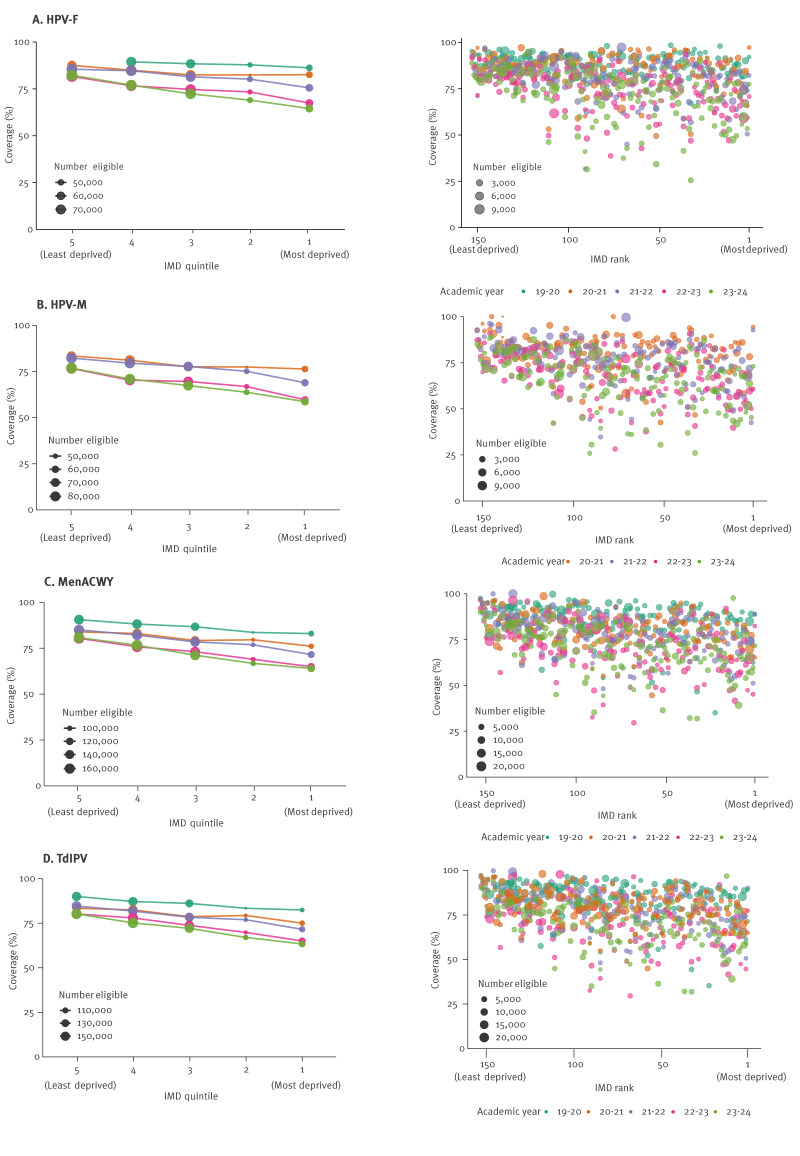
Coverage by IMD quintile and vaccine programme, and coverage by IMD rank and vaccine programme, England, 2017–2024 (n = 2,788)

Figure 2 visualises the relationship between coverage and deprivation across years. The quintile plot illustrates a widening gap in vaccine coverage between the most and least deprived quintiles over time.

F: females; HPV: human papillomavirus; M: males; MenACWY: meningococcal group A, C, W and Y vaccine; TdIPV: tetanus, diphtheria and inactivated polio virus vaccine.

**Figure 3:**
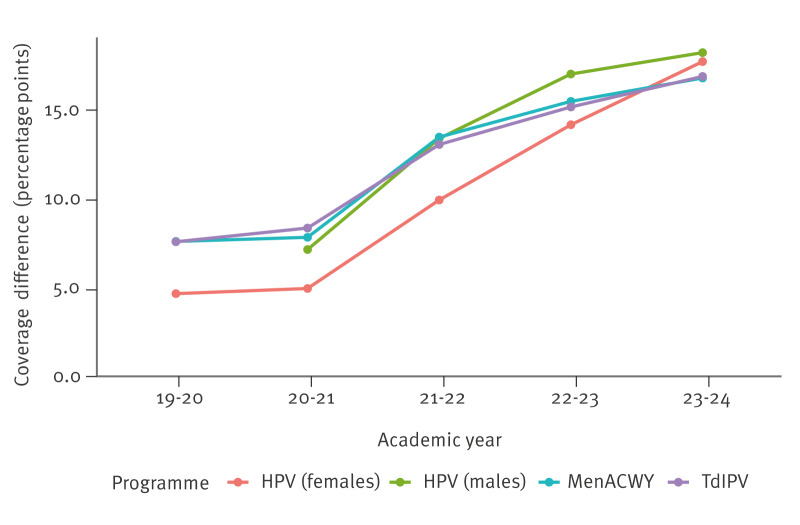
Difference in vaccine coverage between index of multiple deprivation quintiles (Q5–Q1) over time, by programme, England, 2019–2024 (n =1,115)

HPV: human papillomavirus; MenACWY: meningococcal group A, C, W and Y vaccine; TdIPV: tetanus, diphtheria and inactivated polio virus vaccine.

The difference in coverage between the least and the most deprived areas more than doubled between 2019–20 and 2023–24 across all vaccination programmes (Table 3, Figure 3). We formally tested for this increasing difference and found evidence of an increasing difference in coverage between first and last IMD quintile over the time period (likelihood ratio test p < 0.001). We append the full results in Supplementary Table S1. The findings of the London-excluded subgroup analysis are provided in Supplementary Table S2; they broadly mirrored those from the main analysis.

**Table 3:** Effect of index of multiple deprivation on vaccine coverage over time, England, 2019–2024 (n = 1,115)

Vaccine	Coverage in academic year 2019–20 (2020–21 for HPV-M)			Coverage in academic year 2023–24		
	1st IMD quintile	5th IMD quintile	Difference (% points)	1st IMD quintile	5th IMD quintile	Difference (% points)
HPV-F	86.26	90.92	4.66	64.56	82.28	17.72
HPV-M	76.36	83.50	7.14	58.66	76.88	18.22
MenACWY	83.02	90.62	7.60	64.04	80.84	16.80
TdIPV	82.47	90.05	7.58	63.39	80.28	16.89

F: females; HPV: human papillomavirus; IMD: index of multiple deprivation; M: males; MenACWY: meningococcal group A, C, W and Y vaccine; TdIPV: tetanus, diphtheria and inactivated polio virus vaccine.

Q5 = least deprived, Q1 = most deprived).

## Discussion

This analysis provides evidence of a statistically significant association of lower coverage with greater deprivation for all adolescent vaccination programmes since COVID-19, contrasting with some earlier findings that showed no significant area-level disparities in routine HPV vaccination coverage during the initial years of the programme (2008–2011), a period that also saw higher overall coverage [15]. The picture of lower uptake among more deprived socio-economic groups in this analysis from England adds to a growing body of literature frequently – but not universally – reporting associations between low socio-economic status and low uptake. For the HPV vaccine, this includes work from comparator countries such as France, Italy, the Netherlands and the United States [24-27], although close comparison of findings between countries should be undertaken with care given variations in recommended age of first vaccination offer, dosing schedules, service delivery models and wider health system contexts.

Importantly, findings reported here indicate that that inequalities in uptake by deprivation intensified over time in the post-pandemic period in England, in a context of declining coverage overall. This intensification of deprivation-related effects on uptake over time has also been shown for the routine childhood programmes in England [17], although overall trends appear to mask a complex local picture in which considerable variation can be seen between geographies with similar deprivation profiles [28], indicating a need for further investigation. In contrast to findings reported here, analyses of the short-term impact of the pandemic on adolescent HPV vaccination uptake in countries such as Canada [29] and the United States [30] did not find clear evidence of statistically significantly widening gaps between most and least deprived groups, although the outcomes of interest in these studies varied (one focusing on vaccine administration volumes rather than coverage for example).

While adolescent uptake for all three vaccines addressed in this paper remains high by international standards, our findings underscore the importance of sustained action to address inequities in adolescent vaccine uptake across populations. While invasive meningococcal disease in the UK is now at a historically low level [31], this depends on sustaining high vaccination uptake rates. Similarly, adolescent Td/IPV vaccination is designed to provide lifelong protection for young people, and detection of vaccine-derived poliovirus in environmental samples in London in 2022 highlighted again the need for continued vigilance and action to optimise protection in England. While HPV vaccination has had a positive impact on long-standing inequalities in cervical cancer incidence and in cervical screening uptake [31], inequalities in HPV vaccination uptake may undermine this progress [32]. Our findings have longer-term implications in that future cervical screening strategies may be adapted to take into account cohorts vaccinated against HPV. Inequalities in HPV vaccine uptake may also increase inequalities in the incidence of HPV-related oropharyngeal cancer, one of the cancers with the most rapidly rising incidence in high-income countries [32].

The national decline in vaccination coverage across all four programmes since 2020 probably reflects at least in part the impact of the COVID-19 pandemic on school-based vaccination delivery. However, the sustained nature of this decline suggests other contributing factors and merits further investigation. 

The combination of drivers for increasing inequality in adolescent vaccination coverage over time in England is unclear. While declines in overall uptake have been observed over time for routine childhood immunisation in England [33], these have been sustained over 10 years or more and therefore differ from the picture of decline seen principally during and since the pandemic for adolescent vaccination. Contributory factors may include: an inequitable distribution of disruptive effects from the pandemic on school attendance, greater strain on resourcing (human, material and other) to support school-based delivery in areas of high socio-economic deprivation given the intersection of deprivation with factors such as lower literacy and digital exclusion, changing attitudes to vaccination and challenges to securing informed consent (increasingly via digital methods). A combination of these factors is most likely, as for example school non-attendance alone, although increased, is not sufficient to account for the drops in coverage [19].

There are limitations to this analysis, the most important is that data were collated at local authority (ecological) level. We cannot draw clear inferences from these findings regarding factors contributing to uptake at individual level, and further research will be needed to explore, among others, the contribution of healthcare access limitations, variations in vaccination confidence, and logistical challenges in school-based immunisation delivery. Secondly, we used the most recent IMD data, for 2025, across the study period, during which some local authorities may have changed in rank and/or quintile, possibly making the earlier years’ ranking less reliable. However, we also ran this analysis with IMD for 2019, with very similar findings. Furthermore, IMD is a relative and ranked measure of deprivation and does not capture the extent of absolute deprivation. This may fail to adequately capture the effect of absolute deprivation on vaccination coverage.

Our findings have important implications for programme implementation. School-based vaccine delivery remains a powerful mechanism for relatively equitable access, and attitudinal data continue to suggest that confidence both in vaccination and in established delivery mechanisms for adolescents is high [34]. However, disparities in coverage suggest that sustained action will be needed across a range of areas such as awareness raising and stimulating demand for vaccination, measures to increase consent to vaccination, improved access to school-based delivery, and strengthened catch-up activities [35,36]. 

## Conclusion

Although adolescent vaccination uptake in England remains comparatively high by international standards, declining coverage and widening differences between more and less deprived areas pose a growing public health concern and risks widening inequalities in vaccine preventable infections and HPV-related cancers. Targeted catch-up activities could return considerable benefit if designed to deliver vaccination to those living in more deprived areas. There is also a need to improve understanding of the individual and area-level factors contributing to coverage and whether these factors are similar or different across geographical areas. Strengthening programme implementation will therefore require further engagement with schools and local authorities to understand and act on the drivers of low vaccine coverage.

## Data Availability

All data used in this study are derived from publicly available sources: (i) Annual vaccine coverage data from the UK Health Security Agency published on: https://www.gov.uk/government/collections/vaccine-uptake and (ii) English Indices of Deprivation 2025 from the Ministry of Housing, Communities and Local Government published on: https://www.gov.uk/government/statistics/english-indices-of-deprivation-2025. No individual-level data were collected. A collated dataset linking local authority coverage with IMD scores was created using data from the above and can be made available upon request.

## Supplementary Data

265000 Supplement
