# Supplementary material for: Inequalities in adolescent HPV, Td/IPV and MenACWY vaccination coverage by socio-economic status: an ecological study, England, 2017 to 2024
Source: Euro Surveill. 2026 Apr 2;31(13):2500586. doi: 10.2807/1560-7917.ES.2026.31.13.2500586 (PMC13074194; doi:10.2807/1560-7917.ES.2026.31.13.2500586)
Supplement: Supplement [file 25-00586_ALVI_Supplement.pdf]

Appendix

This supplementary material is hosted by *Eurosurveillance* as supporting information alongside the article [Inequalities in adolescent HPV, Td/IPV and MenACWY vaccination coverage by socio-economic status: an ecological study, England, 2017 to 2024], on behalf of the authors, who remain responsible for the accuracy and appropriateness of the content. The same standards for ethics, copyright, attributions and permissions as for the article apply. Supplements are not edited by *Eurosurveillance* and the journal is not responsible for the maintenance of any links or email addresses provided therein.

1. Statistical methods

We used beta regression with a logit link with coverage (ranging from 0 to 1) as a dependent variable and IMD quintile and academic year as explanatory variables. To accommodate coverage values of exactly 100% (reported in 7 out of 3834 UTLA returns) we applied the transformation suggested by Smithson and Verkuilen (2006): $y' = [y*(n-1)+0.5]/n$ where y is the original proportion and n is the sample size. To account for different population sizes served by different UTLAs, we weighted regressions by the number of people eligible for each vaccine programme in each UTLAs.

We formally tested whether the effect of IMD on vaccination coverage changed over time by including in the model an interaction term between IMD quintile and academic year.

2. Results from the model with interaction term

Vaccine	Effect	Coefficient	p-value	lr test
HPV-F	Intercept	2.984 (2.975-2.993)	<0.001	
	Academic year (Q1)	-0.306 (-0.308--0.305)	<0.001	

	IMD quintile 2	0.145 (0.132-0.158)	<0.001	<0.001
	IMD quintile 3	-0.062 (-0.074--0.050)	<0.001	
	IMD quintile 4	-0.203 (-0.215--0.191)	<0.001	
	IMD quintile 5	-0.214 (-0.226--0.202)	<0.001	
	Quintile 2 × year	0.016 (0.014-0.018)	<0.001	
	Quintile 3 × year	0.060 (0.058-0.062)	<0.001	
	Quintile 4 × year	0.104 (0.102-0.105)	<0.001	
	Quintile 5 × year	0.131 (0.129-0.133)	<0.001	
HPV-M	Intercept	2.313 (2.301-2.324)	<0.001	
	Academic year (Q1)	-0.258 (-0.260--0.256)	<0.001	
	IMD quintile 2	0.085 (0.068-0.103)	<0.001	<0.001
	IMD quintile 3	-0.098 (-0.114--0.082)	<0.001	
	IMD quintile 4	0.001 (-0.015-0.016)	0.947	
	IMD quintile 5	-0.123 (-0.139--0.107)	0.102	
	Quintile 2 × year	0.023 (0.020-0.025)	0.022	
	Quintile 3 × year	0.066 (0.064-0.068)	<0.001	
	Quintile 4 × year	0.066 (0.064-0.068)	<0.001	
	Quintile 5 × year	0.115 (0.113-0.118)	<0.001	
MenACWY	Intercept	2.252 (2.246-2.258)	<0.001	
	Academic year (Q1)	-0.222 (-0.223--0.221)	<0.001	
	IMD quintile 2	0.294 (0.285-0.303)	<0.001	<0.001
	IMD quintile 3	0.116 (0.108-0.124)	<0.001	
	IMD quintile 4	0.334 (0.326-0.342)	<0.001	
	IMD quintile 5	0.254 (0.246-0.262)	<0.001	
	Quintile 2 × year	-0.017 (-0.019--0.016)	<0.001	
	Quintile 3 × year	0.028 (0.027-0.030)	0.499	
	Quintile 4 × year	0.028 (0.027-0.029)	0.956	
	Quintile 5 × year	0.068 (0.067-0.069)	<0.001	
TdIPV	Intercept	2.254 (2.248-2.261)	<0.001	
	Academic year (Q1)	-0.220 (-0.221--0.219)	<0.001	
	IMD quintile 2	0.309 (0.299-0.318)	<0.001	<0.001

	IMD quintile 3	0.072 (0.063-0.081)	<0.001	
	IMD quintile 4	0.286 (0.277-0.295)	<0.001	
	IMD quintile 5	0.346 (0.337-0.355)	<0.001	
	Quintile 2 × year	-0.015 (-0.016--0.013)	<0.001	
	Quintile 3 × year	0.041 (0.040-0.043)	<0.001	
	Quintile 4 × year	0.043 (0.042-0.044)	<0.001	
	Quintile 5 × year	0.062 (0.060-0.063)	<0.001	

Table S1: Parameter estimates of Beta regression with interaction term between IMD quintile and academic year and results from likelihood ratio test (lr test)

3. Sensitivity analysis of parameter estimates excluding London boroughs

Vaccine	Effect	OR (95%CI)	p-value
HPV-F	IMD quintile (2nd vs 1st)	1·54 (1·54–1·54)	<0.001
	IMD quintile (3rd vs 1st)	1·57 (1·56–1·57)	<0.001
	IMD quintile (4th vs 1st)	1·67 (1·66–1·67)	<0.001
	IMD quintile (5th vs 1st)	1·92 (1·92–1·93)	<0.001
	Academic year	0·79 (0·79–0·79)	<0.001
HPV-M	IMD quintile (2nd vs 1st)	1·47 (1·46–1·47)	<0.001
	IMD quintile (3rd vs 1st)	1·57 (1·57–1·57)	<0.001
	IMD quintile (4th vs 1st)	1·64 (1·63–1·64)	<0.001
	IMD quintile (5th vs 1st)	1·98 (1·98–1·99)	<0.001
	Academic year	0·82 (0·82–0·82)	<0.001
MenACWY	IMD quintile (2nd vs 1st)	1·36 (1·36–1·36)	<0.001
	IMD quintile (3rd vs 1st)	1·53 (1·53–1·53)	<0.001
	IMD quintile (4th vs 1st)	1·75 (1·75–1·75)	<0.001
	IMD quintile (5th vs 1st)	2·04 (2·04–2·04)	<0.001
	Academic year	0·83 (0·83–0·83)	<0.001

TdIPV	IMD quintile (2nd vs 1st)	1·42 (1·41–1·42)	<0.001
	IMD quintile (3rd vs 1st)	1·58 (1·57–1·58)	<0.001
	IMD quintile (4th vs 1st)	1·88 (1·88–1·88)	<0.001
	IMD quintile (5th vs 1st)	2·17 (2·17–2·18)	<0.001
	Academic year	0·83 (0·83–0·83)	<0.001

Table S2. Parameter estimates of Beta regression, excluding London boroughs*

* Barking and Dagenham, Barnet, Bexley, Brent, Bromley, Camden, Croydon, Ealing, Enfield, Greenwich, Hackney, Hammersmith And Fulham, Haringey, Harrow, Havering, Hillingdon, Hounslow, Islington, Kensington And Chelsea, Kingston Upon Thames, Lambeth, Lewisham, Luton, Merton, Newham, Richmond Upon Thames, Southwark, Sutton, Tower Hamlets, Wandsworth, Westminster.
